# 

**Published:** 1887-07-09

**Authors:** 


PRICE ONE PENNY.

No. 41, Vol. II.
July 9th, 1887.
[Registered at the General Post Office as a Newspaper.]

Per Annum, 6s. 6d., post free.
Monthly Parts, 6d.; post free, 7½d.
Ditto per Ann., 7s. 6d. post free.

### THE HOSPITAL

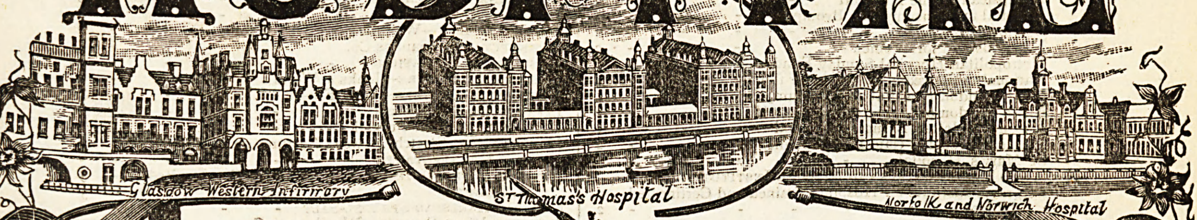

An Institution, Family, and Parochial Journal of Hospitals, Asylums, & all Agencies for the Care of the Sick, Criticism & News.

